# Genomic profiling of native R loops with a DNA-RNA hybrid recognition sensor

**DOI:** 10.1126/sciadv.abe3516

**Published:** 2021-02-17

**Authors:** Kang Wang, Honghong Wang, Conghui Li, Zhinang Yin, Ruijing Xiao, Qiuzi Li, Ying Xiang, Wen Wang, Jian Huang, Liang Chen, Pingping Fang, Kaiwei Liang

**Affiliations:** 1 Department of Pathophysiology, School of Basic Medical Sciences, Wuhan University, Wuhan, China.; 2 Department of Immunology, School of Basic Medical Sciences, Wuhan University, Wuhan, China.; 3 Hubei Key Laboratory of Cell Homeostasis, College of Life Sciences, Wuhan University, Wuhan, P.R. China.; 4 Department of Pharmacology, School of Basic Medical Sciences, Wuhan University, Wuhan, China.; 5 Hubei Province Key Laboratory of Allergy and Immunology, School of Basic Medical Sciences, Wuhan University, Wuhan, China.; 6 Research Center for Medicine and Structural Biology, School of Basic Medical Sciences, Wuhan University, Wuhan, China.

## Abstract

An R loop is a unique triple-stranded structure that participates in multiple key biological processes and is relevant to human diseases. Accurate and comprehensive R loop profiling is a prerequisite for R loops studies. However, current R loop mapping methods generate large discrepancies, therefore an independent method is in urgent need. Here, we establish an independent R loop CUT&Tag (Tn5-based cleavage under targets and tagmentation) method by combining CUT&Tag and GST-His_6_-2×HBD (glutathione *S*-transferase–hexahistidine–2× hybrid-binding domain), an artificial DNA-RNA hybrid sensor that specifically recognizes the DNA-RNA hybrids. We demonstrate that the R loop CUT&Tag is sensitive, reproducible, and convenient for native R loop mapping with high resolution, and find that the capture strategies, instead of the specificity of sensors, largely contribute to the disparities among different methods. Together, we provide an independent strategy for genomic profiling of native R loops and help resolve discrepancies among multiple R loop mapping methods.

## Teaser

A DNA-RNA hybrid recognition sensor protein profiles native R loop structures in the genome.

## INTRODUCTION

An R loop is a special triple-stranded nucleic acid structure formed when nascent RNA invades double-stranded DNA (dsDNA) during transcription, resulting in a DNA-RNA hybrid and a displaced single-stranded DNA (ssDNA). R loops are widely present from bacteria to mammals (*1*). Although R loops have been considered as mere “by-products” of transcription, growing evidence shows that they participate in various key biological processes, such as genome stability maintenance (*1*), transcriptional regulation (*2*, *3*), DNA damage repair (*4*, *5*), and regulation of chromatin landscape (*6*, *7*). Recently, dysfunction in regulation of R loops has been shown to associate with multiple human diseases, including cancers, neurological diseases, and immune disorders (*8*, *9*).

Studying the functions and regulation of R loops in physiological and pathological processes relies on accurate and comprehensive profiling of R loops in the human genome (*10*). During the past decade, several genome-wide R loop mapping methods were developed, using either the S9.6 monoclonal antibody (mAb) or catalytically inactive ribonuclease (RNase) H1 for specific DNA-RNA hybrid (a defining feature of R loops) binding and capturing (*11*). Currently, the predominant strategy for genome-wide profiling of R loops is the DNA-RNA immunoprecipitation sequencing (DRIP-seq), which captures DNA-containing R loop fragments using the S9.6 antibody before sequencing (*12*–*17*). DRIPc-seq is derived from DRIP-seq and specifically sequences the RNA component of the hybrid (*18*). However, the specificity of the S9.6 antibody has been questioned recently for accurate quantification and mapping of genuine R loops (*11*, *19*, *20*). Moreover, the digestion efficiency and bias in chromatin fragmentation by restriction enzymes could potentially compromise R loop mapping resolution in DRIP-related approaches (*21*).

R loop chromatin immunoprecipitation (R-ChIP) takes advantage of the natural affinity of RNase H1 to DNA-RNA hybrids (*22*). An exogenous catalytically inactive RNase H1 was expressed intracellularly to bind DNA-RNA hybrids without resolving them. The DNA (*23*, *24*) or the RNA component (RR-ChIP) (*25*) of the hybrids was sequenced by ChIP assays with V5-tagged RNase H1. R-ChIP/RR-ChIP provides a new perspective of R loop mapping. However, intracellular expression of exogenous catalytically inactive RNase H1 is time consuming, and this mutant RNase H1 could compete with endogenous enzymes for DNA-RNA hybrid binding, which may affect the native R loop status. Alternatively, catalytically inactive RNase H1 was combined with affinity pulldown assays [DNA-RNA in vitro enrichment coupled to sequencing (DRIVE-seq)] or cleavage under targets and release using nuclease (MapR) to map the R loops (*12*, *26*). Nevertheless, DRIVE-seq is less sensitive than DRIP-seq, and its application is limited (*8*, *12*).

R loops participate in multiple biological processes, especially transcription elongation. R loops have been shown to impair transcription elongation by functioning as roadblocks, and R loops are also correlated with transcription pausing near gene promoters (*24*, *27*, *28*). Since dysfunction of R loops and transcription elongation control are implicated in human diseases (*29*), precise and comprehensive mapping of R loops is crucial for studying the functions and mechanisms of R loops in these diseases. Different R loop mapping methodologies have been shown to generate large discrepancies in R loop profiling, especially the genomic distribution of R loops (*8*, *9*, *11*, *23*). For example, R-ChIP and MapR (*26*) both use catalytically inactive RNase H1 and show that R loops are condensed at promoters and almost absent at the 3′ end of genes, whereas DRIP-seq and its derivatives show appreciable signals starting at 2-kb downstream of gene promoters, and much higher signals in the gene body and the 3′ end of genes. The disparities may be caused by the different specificities of RNase H1 and S9.6 to R loops or the different R loop capture and sequencing strategies, such as R loop capture in situ or ex vivo. Moreover, the recombinant catalytically inactive full-length RNase H1 is not very efficient in affinity pulldown (*8*, *12*), which is the principle of DRIP-related R loop mapping methods. Therefore, R loop mapping methodology independent of S9.6 or catalytically inactive RNase H1 is urgently needed to clarify the controversies.

The N-terminal hybrid-binding domain (HBD) of RNase H1 is a short-protein domain, composed of a three-stranded antiparallel β sheet and two short helices. HBD mediates the specific recognition of DNA-RNA hybrids in a nonsequence-specific manner (*22*), which highlights the domain itself as a potential sensor for R loop mapping. Recently, Tn5 transposase was reported to randomly bind DNA-RNA hybrids and transpose adapters onto both strands of the DNA-RNA hybrids (*30*, *31*). Besides, the transposed products could have the strand displaced and be directly used for sequencing library preparation, which save many time-consuming steps (*30*, *31*). Using the Tn5 system for DNA and DNA-RNA hybrids tagmentation would potentially avoid fragmentation bias caused by restriction enzyme digestion.

To test the possibility of using the HBD and Tn5 transposase for R loop profiling, we constructed two glutathione *S*-transferase (GST)–tagged and hexahistidine (His_6_)–tagged artificial sensor proteins (GST-His_6_-HBD and GST-His_6_-2×HBD) with tandem repeats of HBD and established an independent system for native R loop mapping. First, we found that the HBD-containing sensor proteins exhibit high specificity to DNA-RNA hybrids compared to other nucleic acid structures in vitro and GST-His_6_-2×HBD protein behaves similarly to the S9.6 antibody in DRIPc-seq. Furthermore, we combined the R loop sensor protein GST-His_6_-2×HBD or S9.6 with the recently developed Tn5-based cleavage under targets and tagmentation (CUT&Tag) technology (*32*, *33*) and established a native R loop mapping method called R loop CUT&Tag. Compared to conventional R loop mapping methods, R loop CUT&Tag provides superior and highly specific R loop signals at the promoters and is able to detect transient R loops at the gene body and enhancer regions. Together, our study clarifies the controversies among different R loop mapping methods, by providing an independent methodology that accurately and comprehensively profiles the native R loops across the genome.

## RESULTS

### Generation of specific DNA-RNA hybrid sensor proteins with the HBD domain of RNase H1

**Fig. 1:**
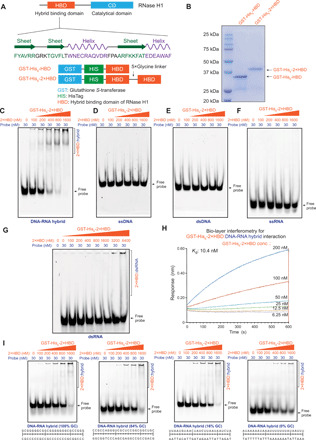
Generation of sensors for specific recognition of DNA-RNA hybrids. (**A**) Schematic depiction of the domain structure of RNase H1 protein. The HBD domain of RNase H1 is responsible for the specific recognition of the DNA-RNA hybrids (*22*). GST-His_6_-HBD and GST-His_6_-2×HBD expression constructs are shown below. (**B**) Analysis of the purified GST-His_6_-HBD and GST-His_6_-2×HBD proteins by SDS–polyacrylamide gel electrophoresis (PAGE) and Coomassie blue staining. (**C** to **G**) EMSAs showing GST-His_6_-2×HBD prefers the DNA-RNA hybrid (C), compared to ssDNA (D), dsDNA (E), ssRNA (F), and dsRNA (G). Fluorescent probes (30 nM) were incubated with increasing concentrations of GST-His_6_-2×HBD (2×HBD) as the indicator for binding. The complexes were resolved with a 6% native polyacrylamide gel and were imaged with a Typhoon FLA-9500. GST-His_6_-2×HBD: DNA-RNA hybrid complexes are indicated by a bracket. (**H**) Biolayer interferometry assay of DNA-RNA hybrid and GST-His_6_-2×HBD. Biotinylated DNA-RNA hybrid was immobilized on streptavidin biosensors and incubated with a range of GST-His_6_-2×HBD (from 6.25 to 200 nM) to measure the response in an Octet Red96 instrument. (**I**) EMSAs analysis of GST-His_6_-2×HBD with probes of different GC contents.

In an attempt to overcome the limits of the S9.6 antibody and catalytically inactive full-length RNase H1, we took advantage of the DNA-RNA hybrid binding proprieties of the N-terminal HBD domain of RNase H1 (*22*), which contains a three-stranded antiparallel β sheet and two short helices (Fig. 1A). For specific DNA-RNA hybrid recognition in vitro, we designed two GST- and His_6_-tagged sensor proteins (GST-His_6_-HBD and GST-His_6_-2×HBD) with tandem repeats of HBD separated by a flexible 5×Glycine linker (Fig. 1A). These proteins were expressed in T7 Express *lysY/I^q^* bacteria cells and were affinity-purified by Ni–nitrilotriacetic acid (NTA) agarose beads (Fig. 1B and fig. S1A). To measure the interaction of recombinant sensor proteins with different forms of nucleic acid structures, including ssDNA, dsDNA, single-stranded RNA (ssRNA), double-stranded RNA (dsRNA), and DNA-RNA hybrid, we tested the affinity of recombinant GST-His_6_-HBD (fig. S1, B to F) and GST-His_6_-2×HBD (Fig. 1, C to G) by electrophoretic mobility shift assay (EMSA) analysis using the fluorescently labeled 25-mer probes and the purified sensor proteins.

EMSA analysis showed that both GST-His_6_-HBD (fig. S1, B to F) and GST-His_6_-2×HBD (Fig. 1, C to G) had the highest affinity for DNA-RNA hybrid, yet little affinity for ssDNA, dsDNA, ssRNA, and dsRNA. dsRNA showed weak interaction with GST-His_6_-2×HBD at high concentrations (Fig. 1G). The results demonstrate that the recombinant GST-His_6_-HBD and GST-His_6_-2×HBD proteins preferentially interact with the DNA-RNA hybrid. Quantitative analysis of recombinant proteins with biotin-labeled DNA-RNA hybrid probes by biolayer interferometry revealed a 10.4 nM dissociation constant *K*_d_ for the complexes of GST-His_6_-2×HBD:DNA-RNA hybrid (Fig. 1H), and 16.5 nM for GST-His_6_-HBD:DNA-RNA hybrid complexes (fig. S1G), respectively. Furthermore, we checked the sequence specificity of GST-His_6_-2×HBD with different probes of various guanine-cytosine (GC) contents and found that GST-His_6_-2×HBD did not have obvious GC preference in DNA-RNA hybrids (Fig. 1I). Further quantification of three biological replicates of EMSA experiments using free probes showed that 0% GC substrate had a slightly weaker binding with 1600 nM GST-His_6_-2×HBD than other probes (fig. S1H). We also confirmed that GST-His_6_-2×HBD could bind the three-stranded R loop structure (fig. S1I). Testing GST-His_6_-2×HBD with probes of different lengths in EMSA (fig. S1, J to L) showed that the 25-mer probe had a slightly stronger interaction with GST-His_6_-2×HBD than the 12-mer probe, while the 48- and 25-mer probe had similar binding to GST-His_6_-2×HBD. Together, these data suggest that the recombinant HBD sensor proteins can be potentially used in R loop profiling as specific DNA-RNA hybrid recognition modules. We chose the GST-His_6_-2×HBD for the rest of the studies, because of its slightly higher affinity with the DNA-RNA hybrid.

### GST-His_6_-2×HBD and S9.6 have well-correlated profiles in affinity pulldown-based DRIPc-seq

**Fig. 2:**
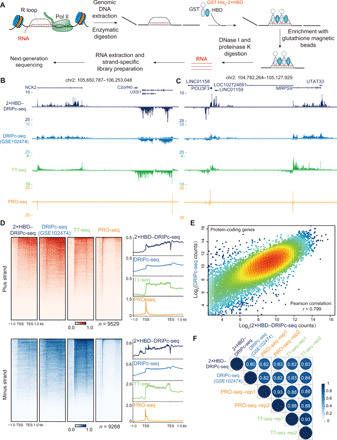
Well-correlated profiles of GST-His_6_-2×HBD and S9.6 in DRIPc-seq–based R loop analysis. (**A**) Schematic presentation of DRIPc-seq with GST-His_6_-2×HBD protein. (**B** and **C**) UCSC genome browser tracks of 2×HBD-DRIPc-seq, DRIPc-seq (GSE102474) (*34*), PRO-seq, and TT-seq reads density at the *NCK2*, *UXS1* (B), *MRPS9*, and *POU3F3* (C) loci. Read density was normalized by reads per million (r.p.m.). (**D**) Heatmap and metagene plots of 2×HBD-DRIPc-seq, the published DRIPc-seq, PRO-seq, and TT-seq signals in the plus and minus strands. (**E**) Scatter plot of the 2×HBD-DRIPc-seq counts and S9.6–DRIPc-seq counts with all of the protein-coding genes. The Pearson correlation coefficient is shown. (**F**) The genome-wide Pearson correlation heatmap of 2×HBD-DRIPc-seq, S9.6-DRIPc-seq, and TT-seq showing densities within all protein-coding genes.

As GST-His_6_-2×HBD specifically bound DNA-RNA hybrid and R loop in vitro, we then investigated whether it could replace the S9.6 antibody in the DRIPc-seq assay, in which DNA-RNA hybrids are immunoprecipitated with the anti–DNA-RNA hybrid S9.6 antibody and the associated RNA molecules are sequenced in a stranded manner to map R loops (*18*). We established a similar DRIPc-seq method with GST-His_6_-2×HBD using the same restriction enzymes, R loop enrichment strategy, and RNA-based library preparation as the S9.6-based DRIPc-seq (Fig. 2A) (*18*). First, we optimized the restriction digestion, amount of GST-His_6_-2×HBD for DNA-RNA hybrid immunoprecipitation, and library preparation for GST-His_6_-2×HBD–based DRIPc-seq (fig. S2, A to C). After optimization, we successfully profiled the genome-wide R loop signals with GST-His_6_-2×HBD as shown in Fig. 2 (B and C) and fig. S2D. Since R loop formation requires transcription elongation of RNA polymerase II (Pol II), we also performed precision nuclear run-on sequencing (PRO-seq) and transient transcriptome sequencing (TT-seq) for genome-wide mapping of the elongating Pol II in human embryonic kidney (HEK) 293T cells (Fig. 2, B and C, and fig. S2D). University of California, Santa Cruz (UCSC) genome browser tracks of our 2×HBD-DRIPc-seq data revealed similar profiles as the published S9.6-based DRIPc-seq (GSE102474) (*34*) at individual genomic loci (Fig. 2, B and C, and fig. S2D).

To compare 2×HBD-DRIPc-seq and S9.6-DRIPc-seq in the genome-wide scale, we plotted the heatmap and metagene plots at all of the protein-coding genes on both minus and plus strands. The results revealed that 2×HBD-DRIPc-seq had well-correlated profiles as S9.6-DRIPc-seq, and the R loop signals mostly localize at the downstream of promoter, gene body, and transcription end site (TES) (Fig. 2D). Quantitative analysis of 2×HBD-DRIPc-seq and S9.6-DRIPc-seq also revealed a high Pearson correlation coefficient of 0.799 (Fig. 2E). Besides, the DRIPc-seq signals generated from both GST-His_6_-2×HBD and S9.6 were positively correlated with elongating Pol II as represented by TT-seq and PRO-seq densities (Fig. 2F and fig. S2, E and F). Since R loops are cotranscriptionally generated, we isolated and sequenced the RNA tethered with chromatin to identify the nascent RNA transcripts (fig. S3A). Genome browser track examples showed that the chromatin-associated RNA sequencing (RNA-seq) profile was similar to GST-His_6_-2×HBD and S9.6-based DRIPc-seq (fig. S3B), and genome-wide analysis of these signals indicated that they were well correlated (fig. S3, C and D). Together, these results demonstrate that the GST-His_6_-2×HBD sensor protein could be used in affinity pulldown assays and exhibits similar profiles as the S9.6 antibody in the DRIPc-seq assays.

### Establishment of a genome-wide native R loop mapping method by CUT&Tag

**Fig. 3:**
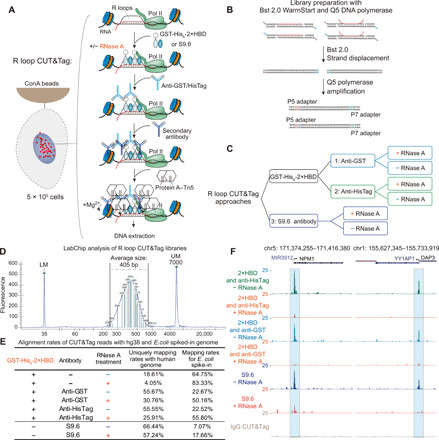
Establishment of the R loop CUT&Tag for native R loop mapping. (**A**) Overview of the R loop CUT&Tag workflow. Cells were immobilized on concanavalin A (ConA)–coated magnetic beads, followed by cell permeabilization. GST-His_6_-2×HBD or S9.6 is used to recognize the R loops in the presence or absence of RNase A. Anti-GST, anti-HisTag, or secondary antibodies were applied to enhance the tethering of pA-Tn5 transposome at the GST-His_6_-2×HBD or S9.6-bound sites. After extensive wash, the pA-Tn5 transposome is activated to integrate the adapters on the chromatin. (**B**) CUT&Tag library preparation with Bst 2.0 WarmStart and Q5 polymerase. Strand displacement was performed with Bst 2.0, followed by library amplification with Q5 DNA polymerase. (**C**) Three different approaches for R loop CUT&Tag analysis. (**D**) LabChip analysis of R loop CUT&Tag library demonstrating the library size ranges from 220 to 700 bp with an average size of 405 bp. UM, upper marker; LM, lower marker. (**E**) Alignment rates of R loop CUT&Tag reads to the human hg38 and *E. coli* spiked-in genomes. RNase A treatment markedly decreases the alignment rates of CUT&Tag reads to the human genome, suggesting the specificity of GST-His_6_-2×HBD and S9.6 on R loop recognition. (**F**) UCSC genome browser tracks of CUT&Tag signals at the *NPM1* and *YY1AP1* loci. The tracks were normalized by reads per million, and the RNase A–treated groups were further normalized with the *E. coli* spike-in control.

The DRIP-based R loop mapping techniques use a combination of restriction enzymes to digest genomic DNA before immunoprecipitation and subsequent next-generation sequencing. The restriction enzymes were reported to be biased and could not fragment the genome uniformly, leading to decreased resolution of DRIP-based R loop profiling (*21*). To avoid potential interference of genomic DNA fragmentation and check the possibility of using GST-His_6_-2×HBD for native R loop profiling, we tested the GST-His_6_-2×HBD sensor protein and the S9.6 antibody with CUT&Tag (*32*, *33*) for in situ and fragmentation-free R loop mapping. First, we constructed and purified a protein A–tethering Tn5 transposase (pA-Tn5) with Ni-NTA affinity purification and performed the pA-Tn5 transposome assembly with adapters (see Materials and Methods). Next, we designed the CUT&Tag workflow for native R loop mapping (Fig. 3A), which used Bst 2.0 WarmStart DNA polymerase for strand displacement followed by library amplification (Fig. 3B). In the CUT&Tag analysis, we designed three different approaches to mapping the native R loops: The first approach used the GST-His_6_-2×HBD and an anti-GST antibody for GST-tagged protein recognition; the second approach also relied on GST-His_6_-2×HBD but required the binding of an anti-HisTag antibody; and the last approach used the S9.6 antibody for R loop detection. The Tn5-based chromatin profiling methods raised concerns of potential tagmentation of accessible DNA, which may generate assay for transposase-accessible chromatin sequencing such as signals (*35*). We used RNase A to evaluate this potential artifact, since the DNA-RNA hybrids are resistant to RNase A digestion at high salt concentrations (>300 mM) and become highly sensitive to RNase A with decreasing salt concentrations. We evaluated all these three approaches with digesting the RNA and DNA-RNA hybrids using RNase A (Fig. 3C).

First, we optimized the Tn5-mediated tagmentation in S9.6-based CUT&Tag with different additives such as 0.85 mM adenosine 5′-triphosphate (ATP), 10% *N*,*N*-dimethylformamide (DMF), and 9% polyethylene glycol, molecular weight 8000, as suggested by a recent Tn5-based DNA-RNA hybrid tagmentation study (*30*). Supplementing 10% DMF markedly enhanced the tagmentation, while the addition of 0.85 mM ATP further improved the effect (fig. S3E). With the improved tagmentation, we generated CUT&Tag sequencing libraries ranging from 220 to 700 base pairs (bp), with the average size of around 405 bp (Fig. 3D). Because of the concomitant *Escherichia coli* genomic DNA during pA-Tn5 transposase protein production, we used the DNA derived from the *E. coli* genome for spike-in normalization, as reported by a previous study (*32*). After alignment of R loop CUT&Tag reads to the human hg38 and the *E. coli* genomes, we noticed that RNase A treatment markedly decreased the alignment rates of CUT&Tag reads to the human genome and increased the percentages of *E. coli* reads with all three different approaches (Fig. 3E). As shown in Fig. 3F and fig. S3F, we detected the R loop signals at the transcription start site (TSS) of the representative genes *NPM1* and *YY1AP1*, as well as R loop signals in the gene body (fig. S3F), while RNase A digestion led to marked reduction of signals at these loci, suggesting that the R loop CUT&Tag signals are not artifacts due to the DNA accessibility.

### Characterization of R loop CUT&Tag signals identified by the GST-His_6_-2×HBD and S9.6 antibody

**Fig. 4:**
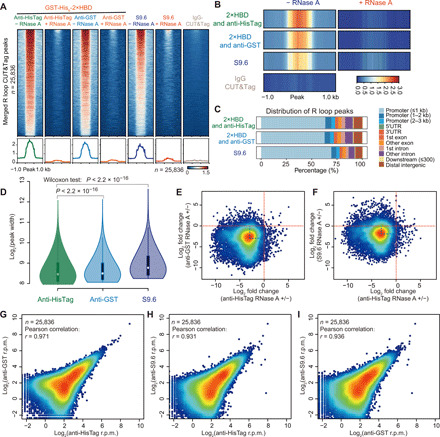
Characterization of native R loops by CUT&Tag with three different approaches. (**A**) Analysis of R loop CUT&Tag signals at all of the peaks from GST-His_6_-2×HBD and S9.6 CUT&Tag. RNase A digestion markedly reduced the CUT&Tag signals at those peaks, suggesting great specificity of GST-His_6_-2×HBD and S9.6 with R loops. (**B**) Heatmap profiles of CUT&Tag signals with or without RNase A treatment. (**C**) Annotation of CUT&Tag peaks showing the localization of the majority of R loops at the promoter regions. The genomic features are shown on the right. UTR, untranslated region. (**D**) Violin plot of CUT&Tag peak width with three different approaches. Wilcoxon test was used to test the statistical differences. CUT&Tag analysis with anti-HisTag antibody and GST-His_6_-2×HBD provides a superior resolution of R loop mapping. (**E** and **F**) Scatter plots of the log_2_ fold changes of R loop signals detected by anti- HisTag with RNase A (+/−) versus the log_2_ fold changes of anti-GST and RNase A (+/−) (E) or log_2_ fold changes of S9.6 and RNase A (+/−) (F). CUT&Tag analysis with anti-HisTag antibody and GST-His_6_-2×HBD is the most specific approach for R loop mapping. (**G** to **I**) Scatter plots of CUT&Tag signals from three different approaches. Pearson correlation was performed, and the *r* values are shown.

To comprehensively compare the three aforementioned approaches for R loop CUT&Tag, we performed peak calling and analyzed the distribution of R loop signals over the human genome. The results revealed a high degree of similarity as shown in the heatmap and metaplot analysis (Fig. 4A). Heatmap profiles of R loop CUT&Tag demonstrated that these R loop signals are highly sensitive to RNase A treatment (Fig. 4B). Most of R loop signals within all three approaches localize at the promoter, while some of these signals could distribute in the gene body and intergenic region (Fig. 4C and table S1). Compared with S9.6, GST-His_6_-2×HBD exhibited narrower peak width in CUT&Tag analysis, indicating a slightly better resolution of GST-His_6_-2×HBD for R loop mapping (Fig. 4D). Moreover, the second approach using the GST-His_6_-2×HBD and an anti-HisTag antibody showed the strongest R loop signals (Fig. 4B) and appeared be the most sensitive approach to RNase A digestion (Fig. 4, E and F). Overall, R loop signals generated from all three different approaches were very similar, and their densities were highly and positively correlated with each other (Fig. 4, G to I). Together, these data demonstrate that both S9.6 and GST-His_6_-2×HBD can be used in R loop CUT&Tag analysis and they generate highly similar native R loop profiles.

**Fig. 5:**
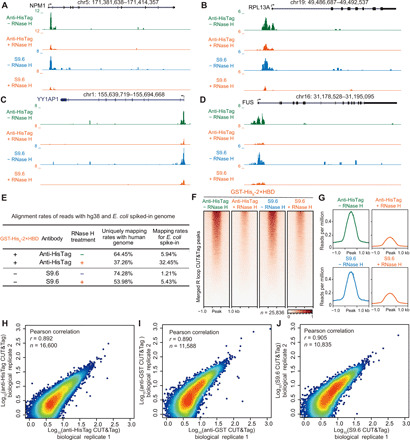
R loop CUT&Tag signals are sensitive to RNase H digestion. (**A** to **D**) UCSC genome browser tracks of CUT&Tag signals at the *NPM1*, *RPL13A*, *YY1AP1*, and *FUS* loci. The tracks were normalized by reads per million and the RNase H–treated groups were further normalized with the *E. coli* spike-in control. (**E**) Alignment rates of R loop CUT&Tag reads to the human hg38 and *E. coli* spiked-in genomes. Four-hour RNase H treatment markedly reduces the alignment rates of CUT&Tag reads to the human genome and increases the alignment rates of reads to *E. coli* spiked-in genomes. (**F** and **G**) Heatmap and metaplot analysis of R loop CUT&Tag signals at all of the peaks from GST-His_6_-2×HBD and S9.6 CUT&Tag. RNase H digestion markedly decreases the CUT&Tag signals at those peaks, demonstrating great specificity of GST-His_6_-2×HBD and S9.6 on R loop recognition. (**H** to **J**) Reproducibility of R loop CUT&Tag methods. Biological replicates were performed, and the Pearson correlation was calculated with the reads per million at R loop peaks.

Furthermore, we tested the sensitivity of R loop CUT&Tag signals to RNase H, which digests DNA-RNA hybrid. As shown in Fig. 5 (A to D), RNase H treatment markedly decreased the R loop signals at individual genes such as *NPM1*, *YY1AP1*, *FUS*, and *RPL13A*. We also found that RNase H treatment decreased the alignment rates of CUT&Tag reads to the human genome and increased the percentages of *E. coli* reads in both GST-His_6_-2×HBD– and S9.6 antibody–based R loop CUT&Tag (Fig. 5E), which had a similar trend as RNase A–treated R loop CUT&Tag (Fig. 3E). Heatmap and metaplot analysis of R loop CUT&Tag signals at all of the peaks from GST-His_6_-2×HBD and S9.6 CUT&Tag confirmed the substantial reduction of R loop signals after RNase H treatment (Fig. 5, F and G), which demonstrates specificity of GST-His_6_-2×HBD and S9.6 in R loop CUT&Tag. We also noticed that RNase H treatment did not completely abolish the CUT&Tag signals, which is consistent with a recent study (*36*) showing that a subset of DNA-RNA hybrids with high GC skew are partially resistant to RNase H. This result may be attributed to the digestion efficiency of commercial RNase H (*18*, *36*) or the potential protection of DNA-RNA hybrids by some R loop binding proteins. Moreover, to measure the reproducibility of R loop CUT&Tag analysis, we performed independent studies at different days and found high reproducible results across independent biological replicates with the R loop CUT&Tag methods (Fig. 5, H to J). Together, these data suggest that GST-His_6_-2×HBD and S9.6 are specific and reproducible for native R loop mapping in CUT&Tag analysis.

### Systematic comparison of R loop CUT&Tag with other conventional R loop mapping methodologies

**Fig. 6:**
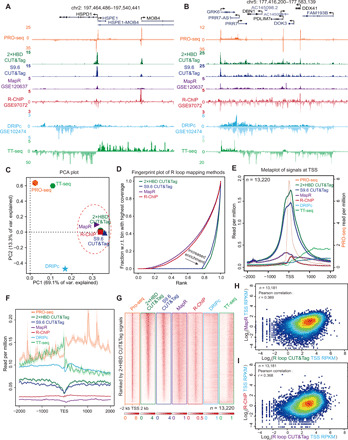
A systematic comparison of R loop CUT&Tag versus other conventional R loop mapping methods. (**A** and **B**) Track examples of HEK293T PRO-seq, GST-His_6_-2×HBD CUT&Tag, S9.6 CUT&Tag, MapR (*26*), R-ChIP (*24*), DRIPc-seq (*34*), and TT-seq signals at the *HSPD1* (A) and *GRK6* (B) loci. The reads were aligned to the human hg38 genome, and the signals were normalized by reads per million. (**C**) PCA plot showing R loop CUT&Tag, MapR, and R-ChIP clustered together. (**D**) Fingerprint plots of R loop CUT&Tag, MapR, and R-ChIP. w.r.t., with respect to. (**E** and **F**) Metaplots of signals detected by different R loop mapping methods, PRO-seq, and TT-seq around the 2-kb window of the TSSs and TESs. Strand-specific signals from PRO-seq, TT-seq, DRIPc-seq, and R-ChIP were used for plotting. (**G**) Heatmap analysis of PRO-seq, TT-seq, and R loop mapping methods at the TSS of transcriptionally active genes (the reads per million of PRO-seq signals at TSS, >1; *n* = 13,220). The heatmaps are sorted by the GST-His_6_-2×HBD CUT&Tag signals. R loop CUT&Tag assays, MapR, and R-ChIP have enrichment at the TSS, while DRIPc-seq does not show this trend. (**H** and **I**) Scatter plots of R loop CUT&Tag and MapR reads per kilobase, per million mapped reads (RPKM) values (H) or R-ChIP RPKM values (I) at TSS. The *r* values were calculated by Pearson correlation.

To systematically compare the R loop CUT&Tag methods with conventional R loop mapping methods, we downloaded the raw data of R loop profiles generated by MapR (*26*), R-ChIP (*24*), and DRIPc-seq (*34*) and realigned them to the human genome hg38. As illustrated in Fig. 6 (A and B), R loop CUT&Tag with GST-His_6_-2×HBD or S9.6 had similar patterns as R-ChIP and MapR, showing concentrated signals at TSS sites, whereas R loop CUT&Tag had much higher overall signal densities. R loop CUT&Tag was different from DRIPc-seq, which distributes highly across the gene body and TES regions. Principal components analysis (PCA) and correlation analysis of these data confirmed that R loop CUT&Tag clustered together with R-ChIP and MapR, and they were distinctly away from DRIPc-seq, PRO-seq, and TT-seq (Fig. 6C and fig. S4A). Moreover, the fingerprint plot of R loop CUT&Tag, R-ChIP, and MapR showed that R loop CUT&Tag had the highest signal-to-noise ratio in R loop profiling (Fig. 6D).

To compare the genome-wide R loop signals among different methods, we selected all of the expressed genes presenting PRO-seq signals at the TSS sites [the reads per million of PRO-seq signals at TSS, >1; *n* = 13,220] in HEK293T cells. Metaplot, metagene plot, and heatmap analysis with these genes showed that CUT&Tag, MapR, and R-ChIP signals were highly concentrated at the TSS sites (Fig. 6, E to G, and fig. S4, B and C). The overall enrichment of R loop signals by CUT&Tag was much higher than other R loop mapping methods including MapR and R-ChIP. In agreement with previous studies (*15*, *23*), we noticed the benefits of R-ChIP for strand-specific R loop mapping, while DRIPc-seq signals distributed across the gene body and TES regions (Fig. 6F and fig. S4, B and C). Besides, we found that the R loop densities at the promoter region generated by CUT&Tag, R-ChIP, and MapR were positively correlated (Fig. 6, H and I). Together, these results demonstrate that R loop CUT&Tag with either GST-His_6_-2×HBD or S9.6 behaves similarly to R-ChIP and MapR but shows much higher R loop enrichment than R-ChIP and MapR at promoter regions. Since these R loop mapping methods use different R loop capture strategies, our data suggest that the capture strategies may substantially affect the R loop profiling.

### Characterization of native R loops at the gene body and enhancer regions

**Fig. 7:**
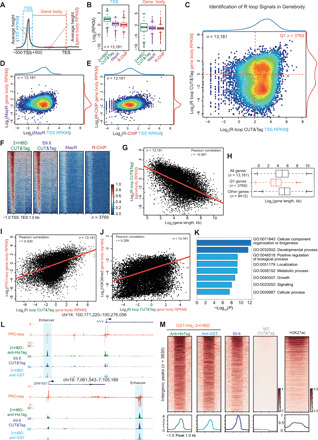
R loop CUT&Tag sensitively detects signals at gene body and intergenic regions. (**A**) Scheme of calculation of signals at TSS and gene body. The signals were normalized by RPKM. (**B**) Box plots of RPKM values at the TSS and gene body from 13,181 transcriptional active genes. (**C**) Scatter plot of R loop CUT&Tag signals at the TSS and gene body showing that R loop CUT&Tag is capable of genome wide detecting the R loop in the gene body. (**D** and **E**) Scatter plots of MapR (D) and R-ChIP (E) RPKM signals at the TSS and gene body. (**F**) Heatmap plots of the 3769 genes with R loop signals at gene body (**G** and **H**) The R loop signals at gene body were negatively correlated with gene lengths. (**I** and **J**) The gene body R loop signals positively correlate with PRO-seq (I) and H3K36me3 (J) signals at the gene body. (**K**) Gene ontology (GO) analysis of the 3769 genes indicates that R loop may be involved in the regulation of various key biological processes. (**L**) Track examples of HEK293T PRO-seq, GST-His_6_-2×HBD CUT&Tag, and S9.6 CUT&Tag signals at the *YY1* and *ZNF557* genomic loci. The reads were normalized by reads per million, and the enhancers are indicated. (**M**) Heatmap analysis of R loop CUT&Tag signals at 3830 intergenic regions. The heatmaps were sorted by the GST-His_6_-2×HBD CUT&Tag signals, and the H3K27ac signals in HEK293T are shown. H3K27ac, histone 3 lysine 27 acetylation.

As shown in Fig. 6 (A and B), R loop CUT&Tag signals were distributed at both the promoter and gene body regions. Next, we performed genome-wide assessment of the R loop signals by calculating the densities [reads per kilobase, per million mapped reads (RPKM)] of R loop at the TSS and gene body regions (Fig. 7A). We successfully calculated 13,181 genes with R loop signals at the TSS sites of the 13,200 expressed genes. Box plots of RPKM from the 13,181 transcriptional active genes showed that TSS densities generated from CUT&Tag, R-ChIP, and MapR were higher than densities at the gene body (Fig. 7B), which is consistent with the metagene analysis (fig. S4B). Furthermore, we noticed the variability of CUT&Tag densities at the gene body regions, suggesting high heterogeneity of densities at gene body among transcriptional active genes as measured by R loop CUT&Tag (Fig. 7B). Therefore, we further plotted the R loop CUT&Tag signals at the TSS and gene body and found that densities at the gene body had a bimodal (two peaks) distribution pattern (Fig. 7C), whereas this bimodal distribution pattern was not observed in R-ChIP or MapR (Fig. 7, D and E). These observations indicate that R loop CUT&Tag is capable of genome-wide detection of R loop. Together, these results suggest that R loop CUT&Tag is more sensitive than R-ChIP and MapR for R loop detection at the gene body regions.

With the aforementioned strategy, we identified 3769 genes (Q1) with R loop densities at the gene body using the cutoffs indicated in Fig. 7C (table S2). Heatmap plots of these 3769 genes confirmed R loop signals at the gene body detected by R loop CUT&Tag (Fig. 7F). We further analyzed the genomic features of the 3769 genes and found that these genes were generally clustered together (Fig. 6B) and associated with short gene lengths (Fig. 7, G and H). Since elongating Pol II is required for R loop formation at gene body, we calculated the PRO-seq and histone 3 lysine 36 trimethylation (H3K36me3) (GSE145160) (*37*) signals at the gene body and found that R loop densities were positively correlated with PRO-seq signals and H3K36me3 densities at gene body (Fig. 7, I and J), suggesting the authenticity of R loop signals at gene body and that these R loops may associate with transcription elongation. Gene ontology analysis of the 3769 genes showed that they were enriched in multiple cellular processes (Fig. 7K) including cellular component organization or biogenesis, developmental process, metabolic process, and cell growth, indicating that R loops at gene body may participate in the regulation of various key biological processes.

Because of the short half-lives of enhancer RNAs and low transcriptional outputs of enhancers, R loops formed at enhancers are likely to be transient and dynamic. However, we still observed R loop CUT&Tag signals at enhancer regions as shown in Fig. 7L. Annotation of R loop CUT&Tag peaks revealed a subset of R loop peaks localized at the intergenic regions (Fig. 4C and table S1). Genome-wide analysis of R loop CUT&Tag peaks identified 3830 intergenic regions, and the heatmap analysis also confirmed that R loop CUT&Tag signals could distribute at these intergenic regions (Fig. 7M). Together, these data indicate that R loop CUT&Tag is sensitive in detecting the transient and dynamic R loops at enhancers.

### R loop profiling is dependent on the ex vivo and in situ capture strategies

**Fig. 8:**
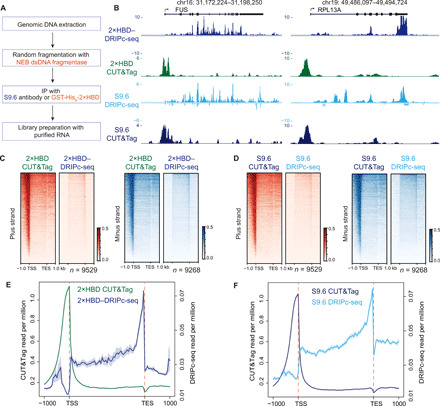
R loop signals are affected by ex vivo and in situ detecting strategies. (**A**) Workflow of DRIPc-seq with GST-His_6_-2×HBD or S9.6 combined with random fragmentation of genomic DNA by NEB dsDNA fragmentase. IP, immunoprecipitation. (**B**) Genome browser tracks of DRIPc-seq and R loop CUT&Tag coverage at the *FUS* and *RPL13A* loci detected by GST-His_6_-2×HBD and S9.6. Signals were normalized by reads per million. (**C** and **D**) Heatmap analysis of DRIPc-seq (ex vivo) and R loop CUT&Tag (native, in situ) at all the protein-coding genes by GST-His_6_-2×HBD (C) or S9.6 (D). (**E** and **F**) Metagene plots of DRIPc-seq and R loop CUT&Tag by GST-His_6_-2×HBD (E) or S9.6 (F).

As shown in Fig. 6 and fig. S4, the ex vivo or in situ capture strategies may substantially affect the R loop profiling in the genome. To systematically compare the difference between the ex vivo and in situ capture strategies, we established a modified DRIPc-seq method with random fragmentation by the New England Biolabs (NEB) dsDNA fragmentase to avoid the bias of restriction digestion (Fig. 8A) (*21*). We tested both the GST-His_6_-2×HBD and S9.6 in the modified DRIPc-seq and compared them to R loop CUT&Tag analysis. Genome browser snapshots at the *FUS* and *RPL13A* loci showed that DRIPc-seq signals appeared downstream of the TSS sites and distributed mainly at the gene body and TES regions (Fig. 8B). Instead, R loop CUT&Tag used the in situ capture strategy and could capture the high signals at the promoter regions and some signals at the gene body and TES. The promoter-associated R loop signals most likely come from the paused RNA polymerases, which localize at the downstream of TSS sites. Genome-wide analysis of R loop signals by the GST-His_6_-2×HBD and S9.6 in DRIPc-seq and CUT&Tag (Fig. 8, C to F) confirmed the global profiling differences between the ex vivo and in situ capture strategies.

Both GST-His_6_-2×HBD and S9.6 showed CUT&Tag signals upstream of the TSS sites. Compared to the DRIPc-seq method, which detects the RNA moiety of DNA-RNA hybrid, the CUT&Tag used the proximal tagmentation strategy and the pA-Tn5 at the binding sites of sensor protein could access the nearby regions and add the adaptors to the nearby DNA and DNA-RNA hybrids. The library size distribution analysis (Fig. 3D) showed that the CUT&Tag libraries had an average size of 405 bp. After deduction of the sequencing adaptors, the insertion fragments are around 269 bp and could cover the regions upstream of TSS and even the downstream of Pol II pausing sites. Another possible explanation for these R loop signals upstream of TSS is the bidirectional transcription at the promoter regions, which has been reported in R-ChIP (*23*), RR-ChIP (*25*) and DRIPc-seq (*15*). We also noticed the difference between GST-His_6_-2×HBD and S9.6 at the TES regions, suggesting that the R loops at TES may have unique characteristics that could be preferentially recognized by the S9.6 antibody in DRIPc-seq. Together, these data showed that the same R loop sensor proteins generated distinct R loop genomic profiling with the ex vivo and in situ capture strategies.

## DISCUSSION

In this study, we constructed specific R loop sensor proteins using the HBD domain of RNase H1 and showed that these sensors bound specifically in vitro to DNA-RNA hybrids and R loops (Fig. 1 and fig. S1), indicating their potential usefulness as new R loop mapping sensors. With GST-His_6_-2×HBD, we established a sensor protein–based DRIPc-seq method that obtained similar R loop profiling to S9.6-based DRIPc-seq (Fig. 2 and fig. S2), showing that GST-His_6_-2×HBD is superior to the catalytically inactive RNase H1 in affinity pulldown assays (*8*, *12*). To eliminate concerns with the bias of fragmentation by restriction enzymes (*21*), we developed alternative strategies for native R loop mapping through establishing a genome-wide R loop CUT&Tag. We compared our system with other conventional R loop methods and investigated the effects of capture strategies on R loop profiling.

R loop CUT&Tag is a sensitive, reproducible, and convenient method to map genome-wide native R loops with high resolution. We tested R loop CUT&Tag with three different approaches and found that the approach combining anti-HisTag antibody and GST-His_6_-2×HBD protein generated a superior resolution and the highest signals in R loop CUT&Tag analysis (Fig. 4). We also systematically compared the R loop signals detected by CUT&Tag methods using MapR, R-ChIP, and DRIPc-seq (Fig. 6 and fig. S4). Our results showed that CUT&Tag, MapR, and R-ChIP signals were concentrated at the TSS sites, while DRIPc-seq did not show this pattern of distribution (Fig. 6). We also found that the overall enrichment of R loop signals by CUT&Tag was much higher than other R loop mapping methods (Fig. 6, E and G). Furthermore, we optimized the DRIPc-seq method with random fragmentation of genomic DNA by dsDNA fragmentase and compared the ex vivo and in situ R loop detection strategies (Fig. 8). Ex vivo DRIPc-seq detected signals predominantly at the gene body and TES regions. This result may be explained by the relatively short and unstable R loops formed at the promoter due to the DNA sequence and topology (*24*, *38*), while the R loops at the gene body and TES may be longer and relatively stable. Besides, RNase H1 was shown to be recruited to the promoter regions, which may degrade R loops and make the R loops at promoter regions highly dynamic (*24*). Moreover, the genomic DNA purification and fragmentation steps during ex vivo R loop capturing may damage the R loops at promoters. All of these factors could lead to the loss of R loop signals at the TSS and result in an enriched R loop profile at gene body and TES regions in DRIPc-seq (Fig. 8). Besides, since the RNA strands of R loops near the promoter are relatively shorter, it is also possible that the less efficiency of complementary DNA conversion and amplification of short RNA molecules caused the lack of signals at TSS in DRIPc-seq. In contrast, R loop CUT&Tag takes advantage of the in situ capture strategy and is able to capture the prominent signals at the promoter regions and some signals at the gene body and TES. In summary, we revealed that even the same R loop sensor proteins could generate distinct R loop profiles in the ex vivo DRIPc-seq and in situ CUT&Tag analysis, suggesting that the R loop capture strategies, instead of the specificity of S9.6 and RNase H1, are the major contributing factors to the discrepancies within different R loop mapping methodologies.

Although R loop CUT&Tag could detect a subset of genes with R loop signals at the gene body, neither R-ChIP nor MapR showed a similar bimodal distribution pattern, suggesting that R loop CUT&Tag is more sensitive than R-ChIP and MapR in R loop detection at the gene body regions (Fig. 7). The enriched key cellular processes (Fig. 7K) indicate that R loops at their gene body regions may participate in the regulation of various key biological processes. These genes were positively correlated with PRO-seq and H3K36me3 signals (Fig. 7, I and J), suggesting that these R loops may associate with transcription elongation. R loops have been shown to impair transcription elongation through functioning as roadblocks for RNA polymerases (*27*, *28*). Besides, R loop induction is correlated with transcriptional pausing and elevated Pol II pausing at TSS, which allows for increased R loop formation (*24*). The misregulation of R loops and transcription elongation has been implicated in cancer and other human diseases. Mechanistic understanding of transcription elongation and R loop regulation is therapeutically relevant (*29*). Although the R loops at enhancers are transient due to the short-lived nature of enhancer RNAs (*39*) and the low transcriptional output of these cis-elements, the R loop CUT&Tag is capable of detecting the R loops at the enhancers (Fig. 7, L and M). R loops at enhancers were reported to promote antisense long noncoding RNA generation, modulate enhancer activities, and regulate cell differentiation and preprogramming (*25*, *39*–*41*). Our R loop mapping method could facilitate the mechanistic studies of enhancer R loops in gene transcription, DNA replication, DNA mutagenesis, enhanceropathies (*42*), as well as lineage specification and pluripotency.

R loop CUT&Tag is relatively easier and more straightforward than other enrichment-based R loop mapping methods such as DRIPc-seq and R-ChIP. It does not need fixation, sonication, restriction digestion, or generation of stable transgenic cell lines, and it only takes less than a day from cell collection to library preparation. The CUT&Tag analysis starts with half a million cells, which is far less than the minimal requirement of DRIPc-seq and R-ChIP methods, and it allows further optimization with even fewer cells. Thus, our method provides possibilities of genome-wide mapping of the native R loops with limited materials. Moreover, the unique characteristics of Tn5 transposase provide great specificity for R loop CUT&Tag analysis. Tn5 has been widely used in sequencing library preparation for rapid processing and low sample input requirement of dsDNA. In addition to its canonical function in dsDNA tagmentation, Tn5 transposase was recently shown to bind and effectively transpose both strands of DNA-RNA hybrids, which can be amplified for library preparation after strand displacement, avoiding many time-consuming steps (*30*, *31*). Since Tn5 transposase does not react with ssRNA or ssDNA, there are no concerns with the possible RNA contamination or the off-target effect of HBD or S9.6 at loci with ssDNA. Tn5 has not been reported to transpose dsRNA, and even Tn5 could potentially react with dsRNA and ligate the adapters to dsRNA, the strands of the products are prevented from being displaced and amplified by Bst 2.0 and Q5 DNA polymerases. RNase A digestion of RNA and DNA-RNA hybrids markedly abolished the R loop CUT&Tag signals (Fig. 4), demonstrating that R loop CUT&Tag signals were not contamination from tagmentation of accessible DNA (*35*). Using RNase H to digest DNA-RNA hybrids during the antibody binding process substantially reduced R loop signals (Fig. 5), indicating the specificity of GST-His_6_-2×HBD and S9.6 in R loop CUT&Tag analysis. Although the current form of R loop CUT&Tag does not provide strand information about R loops, modification of CUT&Tag library preparation to sequence both DNA and RNA strands of R loops is likely to provide useful strand information and better resolution.

## MATERIALS AND METHODS

### Cell culture conditions and DNA construction

HEK293T cells were obtained from American Type Culture Collection and maintained in Dulbecco’s modified Eagle’s media (Life Technologies) supplemented with 10% fetal bovine serum (LONSERA) and 1× penicillin-streptomycin (Life Technologies) at 5% CO_2_ at 37°C. *Drosophila* S2 cells were maintained in Schneider’s medium at 25°C. The cells were routinely tested for mycoplasma contamination with the MycoBlue mycoplasma detector (Vazyme). The HBD coding region RNase H1 was amplified by the Phanta Max Super-Fidelity DNA polymerase (Vazyme) using the ppyCAG-RNASEH1-D210N vector (Addgene #111904) and then cloned into the pET16b vector to produce the pET16b-His_6_-HBD plasmid by Gibson assembly. The His_6_-HBD coding region was further amplified and cloned into the pGEX-2 T vector (Sigma-Aldrich) to generate the pGEX-GST-His_6_-HBD plasmid. A 5×Glycine linker and another copy of the HBD coding region were cloned into the pGEX-GST-His_6_-HBD vector to produce the pGEX-GST-His_6_-2×HBD plasmid. The N-terminal 3×Flag-tagged protein A and Tn5 transposase coding sequence was amplified from the 3×Flag-pA-Tn5-Fl plasmid (Addgene #124601) and subsequently cloned into the pET16b vector to create the His_6_-pA-3×Flag-Tn5 expression plasmid.

### Recombinant GST-His_6_-HBD and GST-His_6_-2×HBD proteins

The pGEX-GST-His_6_-HBD and pGEX-GST-His_6_-2×HBD plasmids were individually transformed into the T7 Express *lysY/I^q^* competent *E. coli* cells (NEB, C3013). The transformed colonies were picked and cultured in the 2×YT medium containing ampicillin (100 μg/ml) at 200 rpm in a 37°C shaker. Protein expression was induced by 0.5 mM isopropyl-β-d-thiogalactopyranoside (IPTG) when the optical density at 600 nm (OD_600_) reached 0.6, and the culture was grown for an additional 5 hours at 200 rpm. Bacterial cell pellets were collected and lysed in 40 ml of HEX buffer [20 mM Hepes-NaOH (pH 7.5), 0.8 M NaCl, 10% glycerol, and 0.2% Triton X-100] supplemented with 1× cOmplete, EDTA-free protease inhibitor cocktails (Roche, 04693132001). Homogenization of lysates was performed with a high-pressure homogenizer at 5.5 MPa for 5 min. The supernatant was collected by centrifuging at 12,000 rpm and 4°C for 30 min. The GST-His_6_-HBD and GST-His_6_-2×HBD proteins were purified with the Ni-NTA beads 6FF (Smart Life Sciences) and eluted with the HEX buffer containing 250 mM imidazole. The eluted proteins were dialyzed against the HEX buffer and were concentrated with an Amicon Ultra-15 centrifugal filter unit (Millipore, UFC901008; 10-kDa cutoff). The GST-His_6_-HBD and GST-His_6_-2×HBD proteins were further analyzed by SDS–polyacrylamide gel electrophoresis (PAGE) and Coomassie blue staining.

### Electrophoretic mobility shift assay

Alexa Fluor 488–labeled DNA oligonucleotides, Cy3-labeled RNA oligonucleotides, and their reverse complement DNA and RNA oligos were synthesized by Sangon Biotech (table S3). Fluorescent probes were generated by annealing the fluorescently labeled oligos with or without their reverse complement oligos. To perform the binding assays, 30 nM probes were incubated with the recombinant GST-His_6_-HBD or GST-His_6_-2×HBD proteins at 25°C for 30 min in the binding buffer [20 mM Hepes-NaCl (pH 7.0), 100 mM NaCl, 5% glycerol, 10 mM dithiothreitol (DTT), and 0.5 mM EDTA] supplemented with sheared salmon sperm DNA (5 μg/ml; Thermo Fisher Scientific, AM9680). The probe and protein probe were resolved in a 6% native polyacrylamide gel in 0.5× tris-borate-EDTA buffer (pH 9.5). The polyacrylamide gels were scanned with a Typhoon FLA-9500 (GE Healthcare) to detect the probe signals. Densitometry of bands was performed using ImageJ.

### Biolayer interferometry assay

Biolayer interferometry assays were performed using an Octet Red96 with streptavidin biosensors (ForteBio, 18-5019). The DNA oligonucleotides were synthesized and labeled with biotin at the 5′ end (5′-[biotin]AGC GTG CCG TGC AAC AAC ATT ACA C-3′). Biotin-labeled DNA oligos were annealed with the reverse complement RNA oligonucleotides RNA-25 to generate a biotin-labeled DNA-RNA hybrid. Kinetic titration series were performed in the interaction buffer [20 mM Hepes-NaOH (pH 7.5) and 200 mM NaCl]. The streptavidin biosensors were hydrated in the interaction buffer for 10 min at 25°C. Following the initial 120-s baseline, the streptavidin biosensors were loaded with the biotin-labeled DNA-RNA hybrid for 300 s. Redundant probes were removed by a 200-s baseline adjustment. To measure the interaction between recombinant proteins and DNA-RNA hybrid, the duration time of association and dissociation was set to 600 s. GST-His_6_-2×HBD proteins were serially diluted from 200 to 6.25 nM and loaded in parallel to measure the binding kinetics with DNA-RNA hybrid. Sensorgrams and sensor signals were analyzed by the ForteBio data analysis software (version 7.1).

### Recombinant pA-Tn5 and pA-Tn5 transposome assembly

The His_6_-pA-3×Flag-Tn5 plasmid was chemically transformed into BL21 (DE3) competent bacteria cells. A single colony was picked and inoculated with 5 ml of LB medium containing ampicillin (100 μg/ml). After overnight culture at 37°C and 200 rpm, 5 ml of culture was transferred to 1 liter of LB medium supplemented with ampicillin (100 μg/ml) and cultured at 37°C and 200 rpm. Once the OD_600_ of the culture reached 0.6, 0.2 mM IPTG was added, and the culture was further induced at 23°C and 200 rpm for 5 hours to induce the pA-Tn5 expression. BL21 *E. coli* was collected and resuspended in 20 ml of HXG buffer [20 mM Hepes-KOH (pH 7.2), 0.8 M NaCl, 10% glycerol, and 0.2% Triton X-100] supplemented with 1× EDTA-free protease inhibitor cocktails and subjected to high-pressure homogenization at 4.1 MPa for 5 min. The supernatant was harvested by centrifuging at 12,000 rpm and 4°C for 30 min, and 0.1 ml of 10% poly(ethyleneimine) solution (Sigma-Aldrich, P3143) was added to precipitate bacterial DNA, which was further removed by centrifugation at 12,000 rpm and 4°C for 10 min. The His-tagged pA-Tn5 was purified with the Ni-NTA beads 6FF (Smart Life Sciences) and eluted with the HXG buffer containing 250 mM imidazole. The eluted pA-Tn5 was dialyzed against 2× dialysis buffer [100 mM Hepes-KOH (pH 7.2), 0.2 M NaCl, 0.2 mM EDTA, 2 mM DTT, 0.2% Triton X-100, and 20% glycerol]. The His-tagged pA-Tn5 was concentrated with an Amicon Ultra-15 centrifugal filter unit (Millipore, UFC903008; 30-kDa cutoff) and diluted 1:1 with 100% glycerol. The purified pA-Tn5 protein was analyzed by SDS-PAGE and Coomassie blue staining and quantified by bicinchoninic acid protein assays.

The pA-Tn5 transposome assembly was performed as described previously (*33*). Briefly, the Tn5MErev (5′-[phos]CTG TCT CTT ATA CAC ATC T-3′), Tn5ME-A (5′-TCG TCG GCA GCG TCA GAT GTG TAT AAG AGA CAG-3′), and Tn5ME-B (5′-GTC TCG TGG GCT CGG AGA TGT GTA TAA GAG ACA G-3′) oligonucleotides were diluted with TE buffer [10 mM tris-HCl (pH 8.0) and 1 mM EDTA] to 400 mM. The mosaic end double-stranded oligonucleotides (Tn5MEDS-A/Tn5MEDS-B) were prepared by mixing equal volume of Tn5MErev with Tn5ME-A or Tn5ME-B and were annealed with a Bio-Rad T100 thermal cycler. To generate pA-Tn5 transposome complex, Tn5MEDS-A, Tn5MEDS-B, and purified pA-Tn5 were mixed at 1:1:1 with the final concentration of 37.5 μM, individually. The mixture was incubated on a three-dimensional rotator at room temperature for 1 hour. The activity of pA-Tn5 transposome was confirmed by tagmentation of plasmid DNA in N-[Tris(hydroxymethyl)methyl]-3-aminopropanesulfonic acid (TAPS)-DMF buffer [10 mM TAPS-KOH (pH 8.3), 5 mM MgCl_2_, and 10% DMF].

### Cleavage under targets and tagmentation

CUT&Tag assays were performed as described previously (*32*, *33*) with some modifications. Briefly, 5 × 10^5^ cells were washed twice in 1.0 ml of wash buffer [20 mM Hepes (pH 7.5), 150 mM NaCl, 0.5 mM spermidine, and 1× protease inhibitors] by gentle pipetting. Ten microliters of concanavalin A–coated magnetic beads (Smart Life Sciences) were activated and then added to 5 × 10^5^ cells with incubation at room temperature for 10 min. The supernatant was removed, and bead-bound cells were resuspended in 100 μl of antibody buffer [20 mM Hepes (pH 7.5), 150 mM NaCl, 0.5 mM spermidine, 1× protease inhibitors, 0.05% digitonin, 0.01% NP-40, and 2 mM EDTA]. Two micrograms of recombinant GST-His_6_-2×HBD protein or S9.6 (anti–DNA-RNA hybrid antibody, clone S9.6; Millipore, MABE1095) was added to incubate with the bead-bound cells by rotating overnight at 4°C. As controls, 10 μg of RNase A (Takara) or 20 U of RNase H (NEB, M0297S) was added during the antibody incubation stage. After brief wash with dig-wash buffer [20 mM Hepes (pH 7.5), 150 mM NaCl, 0.5 mM spermidine, 1× protease inhibitors, 0.05% digitonin, and 0.01% NP-40] twice, the GST-His_6_-2×HBD–treated groups were incubated with anti-HisTag mAb (ABclonal, AE003; 1:100 dilution) or anti–GST-Tag mAb (ABclonal, AE006; 1:100 dilution), individually. The S9.6-treated groups were incubated with rabbit anti-mouse immunoglobulin G (IgG) antibody (final concentration, 10 μg/ml). The antibody incubation was performed in 100 μl of antibody buffer and was incubated at room temperature for 1 hour. Bead-bound cells were briefly washed three times with 200-μl dig-wash buffer to remove the unbound antibodies. The mouse anti-rabbit IgG antibody or rabbit anti-mouse IgG antibody was diluted 100× (final concentration, 10 μg/ml) and incubated with the cells for another 1 hour, followed by washing with 200 μl of dig-wash buffer three times.

A 1:250 dilution of pA-Tn5 adapter complex (~15 μM) was prepared in dig-300 buffer [20 mM Hepes (pH 7.5), 300 mM NaCl, 0.5 mM spermidine, 1× protease inhibitors, and 0.05% digitonin]. One hundred microliters of diluted pA-Tn5 complex was mixed with the bead-bound cells and was rotated at room temperature for 1 hour. Bead-bound cells were washed three times in 200 μl of dig-300 buffer to remove unbound pA-Tn5 protein. Next, cells were resuspended in 40 μl of tagmentation buffer [10 mM TAPS-NaOH (pH 8.5), 10 mM MgCl_2_, 10% DMF, and 0.85 mM ATP] and incubated at 37°C for 1 hour. To stop the tagmentation reaction, 2.25 μl of 0.5 M EDTA, 2.75 μl of 10% SDS, and 0.5 μl of proteinase K (20 mg/ml; Roche) were added and were further incubated at 55°C for 60 min. The tagmentation products were purified with 1× Sera-Mag carboxylate–modified magnetic beads (Sigma-Aldrich, GE24152105050350) and were eluted in 10 μl of 0.1% Tween 20. The eluent was mixed with 10 U of Bst 2.0 WarmStart DNA polymerase (NEB, M0538) in 1× Q5 polymerase reaction buffer and was incubated at 65°C for 30 min to perform the strand displacement reaction. The reaction was then stopped by incubation at 80°C for 20 min. To generate the sequencing libraries, the mixture was mixed with a universal i5 primer and a uniquely barcoded i7 primer and then amplified with the Q5 high-fidelity master mix (NEB, M0492). The libraries were size-selected with 0.56 to 0.85× Sera-Mag carboxylate–modified magnetic beads and subjected to LabChip DNA analysis and Illumina sequencing.

CUT&Tag reads were aligned to the human genome (UCSC hg38) with Bowtie version 1.1.2, allowing only uniquely mapping reads with up to two mismatches (*43*). The aligned reads were normalized to total reads aligned (reads per million). The track files were made with the bamCoverage command from deepTools 3.3.0 (*44*). For spike-in normalization, the reads were also aligned to the *E. coli* genome by Bowtie2 with the options (--end-to-end --very-sensitive --no-overlap --no-dovetail --no-mixed --no-discordant --phred 33 -I 10 -X 700) (*43*). The RNase A– and RNase H–treated groups were normalized to their nontreated counterparts by scale factors. CUT&Tag peaks were called using MACS (model-based analysis of ChIP-seq) version 2.1.2 using default parameters and *q* value cutoff of 1 × 10^−4^ (*45*). The distribution of CUT&Tag peaks was annotated with the R package ChIPseeker. Heatmaps, metaplots, and metagene plots were made for the indicated windows using the average coverage (reads per million) (*46*).

### Transient transcriptome sequencing

TT-seq was performed as described (*47*, *48*) with modifications. A total of 1 × 10^7^ cells were labeled with 400 μM 4-thiouridine (4sU; Sigma-Aldrich, T4509) in a CO_2_ incubator at 37°C for 10 min and were quickly lysed with 4 ml of TRIzol (Invitrogen, 15596018). Total RNA was purified with chloroform extraction and precipitated with isopropyl alcohol and 5 μl of glycogen (20 mg/ml; Roche, 10901393001). The extracted RNA was spiked-in with 4sU-labeled S2 RNA and was further fragmented by base hydrolysis in 0.2 M NaOH (15 min, on ice), neutralized by adding 1× volume of 1 M tris-HCl (pH 6.8) and precipitated with isopropyl alcohol. Biotinylation reaction of 4sU-labeled RNA was carried out in a total volume of 250 μl, containing 100 μg of total RNA, 10 mM Hepes (pH 7.5), 1 mM EDTA, and 5 μg of biotin-XX-MTSEA (Biotium, 90066) dissolved in DMF (final concentration of DMF, 20%) at room temperature for 30 min.

After biotinylation, excess biotin reagents were removed by extraction with chloroform and phase lock gel. RNA supernatant was precipitated with a 1:10 volume of 5 M NaCl and an equal volume of isopropyl alcohol. The RNA pellet was resuspended in 200 μl of RNase-free water. After denaturation of RNA samples at 65°C for 5 min followed by rapid cooling on ice for 5 min, biotinylated RNA was purified using 50 μl of Dynabeads MyOne streptavidin C1 (Thermo Fisher Scientific, 65001). MyOne streptavidin beads were incubated with RNA samples for 15 min with rotation at room temperature. Beads were then washed three times with wash buffer [10 mM tris-HCl (pH 7.4), 1 mM EDTA, 1 M NaCl, and 0.1% Tween 20], followed by one step wash at 65°C. 4sU-RNA was eluted with 100 μl of freshly made 100 mM DTT, followed by a second elution with an additional 100 μl of 100 mM DTT.

The eluted RNA was purified with the Sera-Mag carboxylate–modified magnetic beads and was subjected to strand-specific RNA-seq library preparation. Libraries were made with the NEBNext Ultra RNA Library Prep Kit for Illumina and subjected to next-generation sequencing. TT-seq reads were aligned to the human genome (UCSC hg38) with Bowtie 1.1.2 (*43*), allowing only uniquely mapping reads with up to three mismatches within the 50-bp read. The resulting reads were normalized to total reads aligned (reads per million) for each strand with deepTools 3.3.0 (*44*). The reads coverage at indicated regions were calculated by bedtools multicov (version 2.25.0) or HTSeq-count and were then normalized to total reads aligned.

### Chromatin-associated RNA-seq

Chromatin-associated RNA-seq was performed as described before (*49*). Briefly, 10 million HEK293T cells were harvested via trypsin digestion and were washed twice with 10 ml of ice-cold phosphate-buffered saline. Cells were resuspended in 5 ml of ice-cold NUN buffer [20 mM Hepes (pH 7.9), 7.5 mM MgCl_2_, 0.2 mM EDTA, 300 mM NaCl, 1 M urea, 1% NP-40, 1 mM DTT, RNase inhibitor (20 U/ml), and 1× cOmplete EDTA-free protease inhibitor cocktails (Roche, 04693132001)] with gentle pipetting. The suspension was placed on ice for 10 min and centrifuged at 3000*g* for 5 min at 4°C. The chromatin pellet was further washed with 10 ml of NUN buffer four times and lysed with 1 ml of TRIzol reagent. The lysates were heated to 65°C to dissolve the chromatin pellets, and RNA extraction was performed according to the manufacturer’s instruction. The RNA was polyadenylation depleted with Oligo(dT)25 magnetic beads (NEB, #S1419S) and treated with RNase-free deoxyribonuclease I (DNase I) to remove potential mRNA and genomic DNA contamination. The RNA was then purified with phenol/chloroform extraction and ethanol precipitation. Five hundred nanograms of RNA was further ribodepleted with the Ribo-off rRNA depletion kit (Vazyme, #N406), and library preparation was performed with the NEBNext ultra RNA library prep kit for Illumina. Chromatin-associated RNA reads were aligned to the human genome (UCSC hg38) using Bowtie 1.1.2. For track files, reads were normalized by reads per million. Reads at all of the protein-coding genes were counted by HTSeq-count with the union model.

### Precision nuclear run-on and sequencing

PRO-seq was performed as described previously (*47*, *50*). Briefly, all four biotinylated nucleotides (PerkinElmer) were used at 25 mM for the nuclear run-on reaction. RNA 5′ pyrophosphohydrolase (NEB, M0356S) was used to remove the RNA cap to facilitate the downstream library preparation. PRO-seq reads were mapped to the human genome (UCSC hg38) using Bowtie version 1.1.2, allowing only uniquely mapping reads with up to three mismatches (*43*). Aligned reads were then converted to strand-specific bigwig files with the bamCoverage command from deepTools 3.3.0 (*44*). PRO-seq genome browser track examples show coverage of the entire length of the read for visualization. The read counts at indicated regions were calculated by bedtools multicov (version 2.25.0). The heatmaps were generated with the indicated regions with ngs.plot or the computeMatrix command from deepTools 3.3.0 (*44*).

### DRIP coupled to high-throughput sequencing

DRIPc-seq experiments were performed according to a previously published protocol (*18*) with modifications. A total of 8 × 10^6^ 293 T cells were harvested and resuspended in 1.6 ml of TE buffer including 50 μl of 20% SDS and 5 μl of proteinase K (20 mg/ml). After digestion at 37°C for 12 hours, phenol/chloroform extraction and ethanol precipitation were performed to purify the genomic DNA. The DNA was further digested with NEB restriction enzymes (EcoR I, Ssp I, BsrG I, Xba I, and Hind III) or NEBNext dsDNA fragmentase (NEB, M0348). Eight micrograms of digested genomic DNA was incubated with 100 ng of GST-His_6_-2×HBD protein or 20 μg of S9.6 mAb in DRIP-binding buffer [10 mM NaPO_4_ (pH 7.0), 140 mM NaCl, and 0.05% Triton X-100] at 4°C overnight. The 2×HBD:DNA-RNA hybrid complexes and S9.6:hybrid complexes were purified with 20 μl of glutathione magnetic agarose beads (Thermo Fisher Scientific, 78601) or protein A/G agarose at 4°C for 4 hours with rotation. After extensive washes four times, the nucleic acids were eluted and purified as previously described (*18*).

For DRIPc-seq library preparation, the DNA molecules were digested with NEB DNase I for 1 hour at 37°C, and the RNA molecules were recovered with ethanol precipitation. The libraries were prepared with the NEBNext Ultra Directional RNA Library Prep Kit and subjected to LabChip analysis and next-generation sequencing. DRIPc-seq reads were aligned to the human genome (UCSC hg38) with Bowtie 1.1.2, allowing only uniquely mapping reads with up to two mismatches within the first 50 nucleotides (*43*). The resulting reads were normalized with total reads aligned for visualization. The heatmaps were generated with the indicated regions with the computeMatrix and plotHeatmap commands from deepTools 3.3.0 (*44*). The read numbers at indicated regions were counted by the multicov command from bedtools v2.25.0 or HTSeq-count.

### Quantitative polymerase chain reaction

Quantitative real-time polymerase chain reaction (PCR) was performed in triplicate using a Jena qTOWER G real-time PCR thermal cycler. Primer sequences for all quantitative PCR reactions are *RPL13A* forward (5′-AGG TGC CTT GCT CAC AGA GT-3′), *RPL13A* reverse (5′-GGT TGC ATT GCC CTC ATT AC-3′), *TFPT* forward (5′-TCT GGG AGT CCA AGC AGA CT-3′), *TFPT* reverse (5′-AAG GAG CCA CTG AAG GGT TT-3′), *EGR1* forward (5′-GAA CGT TCA GCC TCG TTC TC-3′), and *EGR1* reverse (5′-GGA AGG TGG AAG GAA ACA CA-3′).

### Statistical analysis

All quantitative results were analyzed with the test indicated in the figure legends, after confirming that the data met appropriate assumptions (normality, homogeneous variance, and independent sampling). All the *P* values are two tailed, and the data are presented as means ± SD. The peak or gene size (*n*) in the heatmaps indicates the number of peaks or genes included. In the violin plots, the white dot indicates the median, and the solid box indicates the interquartile range. The statistical tests were performed with R (version 3.6.1).

## SUPPLEMENTARY MATERIALS

Supplementary material for this article is available at http://advances.sciencemag.org/cgi/content/full/7/8/eabe3516/DC1

## Appendix

View/request a protocol for this paper from *Bio-protocol*.
